# The Nicaraguan pediatric influenza cohort study: design, methods, use of technology, and compliance

**DOI:** 10.1186/s12879-015-1256-6

**Published:** 2015-11-09

**Authors:** Aubree Gordon, Guillermina Kuan, William Aviles, Nery Sanchez, Sergio Ojeda, Brenda Lopez, Lionel Gresh, Angel Balmaseda, Eva Harris

**Affiliations:** Department of Epidemiology, School of Public Health, University of Michigan, Ann Arbor, MI USA; Sócrates Flores Vivas Health Center, Ministry of Health, Managua, Nicaragua; Sustainable Sciences Institute, Managua, Nicaragua; Centro Nacional de Diagnóstico y Referencia, Ministry of Health, Managua, Nicaragua; Division of Infectious Diseases and Vaccinology, School of Public Health, University of California, Berkeley, CA USA

**Keywords:** Nicaragua, Influenza, Cohort study, Methods, Central America, Respiratory, Tropics

## Abstract

**Background:**

Influenza causes substantial morbidity and mortality worldwide, yet few data exist on influenza infection rates in tropical, developing countries. In 2011, we established the Nicaraguan Pediatric Influenza Cohort Study (NPICS) to study the burden and seasonality of influenza in Nicaraguan children. Here we describe the study design, methods, and participation data of the NPICS for 2011–2013.

**Methods/Design:**

A total of 1532 children aged 0 to 12 years were enrolled into the study in 2011, and an additional 401 children were enrolled between 2012 and 2013. Children were provided with all of their medical care through the study, and data on medical visits were recorded systematically. A number of surveys were conducted together with a blood sample annually, including a height and weight measurement, a socio-economic status and risk factor survey, and a breastfeeding survey.

**Discussion:**

Unique features of our study include the customized low-cost, open-source informatics system as well as the development of methods to leverage infrastructure and resources by conducting multiple studies in the same setting while maximizing protocol adherence and quality control. These methods should be useful to others conducting large cohort studies, particularly in low-resource settings.

**Electronic supplementary material:**

The online version of this article (doi:10.1186/s12879-015-1256-6) contains supplementary material, which is available to authorized users.

## Background

Influenza causes substantial morbidity and mortality worldwide, with an estimated 1 billion cases and 3–5 million cases of severe disease annually [1]. In tropical developing countries, much remains to be elucidated about the burden and epidemiology of seasonal influenza. The paucity of data is due to both the lack of resources as well as the relative importance of other diseases in these regions. In addition, influenza wasn’t thought to constitute a major disease burden in the tropics [2]. However, recent studies have found that developing countries have a substantial burden of influenza, particularly in young children [3–8]. To study the seasonality and burden of influenza in Nicaraguan children, we established the Nicaraguan Pediatric Influenza Cohort Study (NPICS).

Large-scale prospective cohort studies are notoriously resource-intensive to conduct. As a result, there are relatively few opportunities to perform cohort studies, and they are often not conducted despite established scientific interest and public health need. In infectious disease research, studies usually focus on a single pathogen or syndrome with occasional subsequent use of samples to investigate other pathogens. Here, we have developed a dual cohort for dengue and influenza. Overall, our study sites in Nicaragua were developed over 27 years of close collaboration with the Ministry of Health, including numerous capacity building activities. The cohort site was first established for dengue research studies in 2004 and later expanded to include influenza and other respiratory viruses [9, 10]. Study researchers have spent 8 years building extensive community infrastructure and developing local skills for influenza research. This led to a first influenza cohort study (2007-2010) and then to the NPICS (2011-present).

The objectives of NPICS are: to estimate the incidence of influenza in Nicaraguan children; to investigate the seasonality of influenza in Nicaragua, a tropical country; to evaluate risk factors for influenza virus infection; and to analyze the genetic relation of viruses circulating in Nicaragua to those circulating in the Northern and Southern hemispheres. In addition, the study aims to build capacity at the local site and disseminate scientific knowledge.

## Methods/Design

### Study site, population and design

The NPICS is conducted in District II of Managua, Nicaragua, at the Health Center Sócrates Flores Vivas (HCSFV). The district is adjacent to Lake Managua and includes neighborhoods ranging from low to middle class, including several areas classified as urban slums. The streets of District II are largely paved, all residents have access to electricity, and city water service varies (0–24 h/day), with most residents reporting ≤8 h of service. Person-density in study neighborhoods included ranges from ≥10,000 to ≤57,000 persons/km^2^, with a majority of participants living in areas with 14,000–20,000 persons/km^2^. The HCSFV includes a clinical laboratory and provides primary medical services to a population of ~61,411. Molecular and serological testing for influenza is performed at the National Virology Laboratory (NVL) of the Centro Nacional de Diagnóstico y Referencia (CNDR), which is a WHO National Influenza Center. Both the CNDR and the HCSFV are part of the Nicaraguan Ministry of Health.

The study population consists of children aged 0-14 years who reside in District II of Managua. Initially the study consisted of children aged 0-12 years, however in 2013 the study was expanded to allow children to continue to participate until 14 years of age. At enrollment, a clinical history, socio-demographic survey and household survey are collected. Each March/April, an annual blood collection is performed on children >6 months of age, a healthy height and weight is recorded, and breast-feeding, socio-demographic, and household surveys are administered.

### Recruitment

To maximize follow-up time of participants and thus information on infection history, initial enrollment of three- to eleven-year-old children was done through a random selection of all participants from our previous influenza cohort study [8]. That study was contiguous with the NPICS and consisted of non-immunocompromised children 2–13 years old within the HCSFV catchment area, who were recruited through house-to-house visits. Additional children ≤2 years were recruited for NPICS through house-to-house visits. Each month, newborn infants aged ≤4 weeks are enrolled into the NPICS. Parents or guardians of participants were briefly informed about the NPICS by study staff. If the parent or guardian indicated interest, study staff read the informed consent form aloud, which the parent or guardian then signed if they elected to have their child participate. Verbal assent was obtained from all children aged six and over. No monetary incentives were offered for participation.

The study consent form consists of three parts: the primary consent form (Part A), consent for future use and storage (Part B), and consent for genetic studies (Part C). To increase compliance with consent protocols, the three consent forms are printed on differently colored paper, enabling both field workers and supervisors to easily identify missing components. To facilitate tracking of consent forms and to minimize data entry errors, all consent forms are barcoded. Consent forms are stored in order of study code to facilitate rapid retrieval, separately from other study documents in locked cabinets, in accordance with good clinical practice (GCP).

### Yearly survey and sample collection

Each March/April, annual surveys are conducted and a blood sample is collected, before the influenza season begins, typically in June. Annual surveys include a socio-economic and household risk factor questionnaire, a breastfeeding questionnaire, height and weight, and a satisfaction questionnaire. Survey data are collected directly into a tablet computer or smart phone, with home factors (i.e., variables that are constant between household members) collected separately from participant-specific factors. Blood samples of 7 ml are collected from cohort participants aged ≥2 years and 3 ml from children ≥6 months and <2 years.

The annual sampling and survey is divided into two phases. In the first phase, all participants are encouraged to come to the HCSFV and are contacted via posted notices throughout the HCSFV, text messaging, and telephone calls. The annual survey and sampling at the HCSFV is divided into 7 stations; registration, identification, informed consent, height-and-weight, surveys, blood sampling, and study gift. To route participants through the stations in an orderly fashion, laminated numbered cards are used, with each station assigned a unique color for their cards. When participants arrive at the HCSFV, they are immediately routed to registration, where they are identified by fingerprint or study ID card and their contact information and basic demographic information is confirmed. Children then continue to the height-and-weight station. Each child is measured without shoes in light clothing on a digital scale for weight (Seca 874, Hamburg, Germany) and using a portable stadiometer for height (Seca 213). Children <2 are measured using a digital baby scale (Detecto 8440, Webb City, MO) and an infantometer (Seca 416). Measurements are taken twice and entered directly into a smart phone or tablet using a custom app, which automatically calculates the difference between the two measurements and prompts the user to take a third measurement if the difference is ≥5 %. Next, participants move to the survey station, where staff administer the study factor surveys (see Table 1). Finally, subjects move to the sampling station for collection of a blood sample.

**Table 1:** Questionnaires and surveys, Nicaraguan Pediatric influenza cohort study, Managua, Nicaragua, 2011–2013

Survey/Case report form	Description
Medical consult form	>80 item case report form completed by study nurses and physicians. Includes data on: height, weight, tympanic temperature, blood pressure, respiratory and heart rates, lower and upper respiratory symptoms, systemic symptoms, gastrointestinal symptoms, and nutritional status
Influenza follow-up form	Collects daily information on 14 signs and symptoms. Filled out by physician at follow-up appointments for all possible influenza cases.
Household annual SES and risk factor survey	Questionnaire completed per household. Includes 31 questions on crowding, water access, socioeconomic level, presence of animals, smoking and cooking inside the house.
Participant annual risk factor survey	44 questions focusing on medical history, socio-demographic information, individual risk factors and vaccination
Height and weight survey	Height and weight are measured twice. If there is >5 % difference between the two measurements, a third measurement is taken.
Breastfeeding survey	9 questions about current and past breastfeeding
Satisfaction survey	12 questions about the quality of the service provided by study staff in different areas (admission, nurses, physicians, clinical laboratory)

In the second phase, study teams are pre-assigned a set of participants (who did not attend the HCSFV in the first phase) to visit, maps of the location of each team’s set are printed, and GPS points and participants’ information are downloaded into mobile devices. Throughout the day, data are transferred from the field staff’s devices to a customized study database. This provides near real-time monitoring of field activities, enabling rapid assessment of progress, efficient reassignment of participant visits when necessary, and effective planning of the next day’s visits [11]. Maps showing location and status of the participants are generated in the team’s smart phones and in a web page using the Google maps API.

### Case surveillance and selection for respiratory virus testing

The primary outcomes of this study are laboratory-confirmed influenza and influenza-like illness. A case of laboratory-confirmed influenza is defined as a positive test for influenza A or B by RT-PCR. The definition of influenza-like-illness used in this study is a fever of 37.8 °C with a cough and/or sore throat. Study participants are provided with all of their primary medical care and laboratory testing free of charge. Study physicians and nurses are available at the HCSFV 24 h/day, 365 days/year. Data on all medical and field visits are collected systematically on standardized data collection forms or directly into tablet computers or smart phones.

Parents of participants agreed to bring their child(ren) in to the HCSFV at the first sign of fever. Upon presentation at the HCSFV, participants identify themselves in the reception area. All participants have a fingerprint scan on file and are issued a barcoded ID card. Confirmation of study participation is performed by scanning either the participant’s study ID card or fingerprint. The participant is then directed to the study area, where a study nurse measures and records their height, weight, and tympanic temperature. Next, the child is examined by a study physician, who records information systematically on the history of illness and current symptoms. Data on >80 variables are collected, including a second tympanic temperature, blood pressure, respiratory and heart rates, lower and upper respiratory symptoms, gastrointestinal symptoms, and nutritional status. The criteria for influenza testing in this study is illness onset within 4 days with a fever or history of a fever, as reported by the parent, and cough and/or onset for children ≥2 years, or fever or reported fever for those <2 years. In addition, any child presenting with clinical pneumonia or severe respiratory illness who is referred to tertiary care is tested for influenza. All children who fulfill the influenza testing criteria are scheduled for follow-up appointments, during which physicians complete an influenza follow-up form that collects information on illness duration and severity.

### Respiratory sample collection

Nasal and oropharyngeal swabs were chosen for respiratory sample collection to minimize risk to participants and increase compliance with repeated sampling, as these are the least invasive ways to collect samples for respiratory virus testing. Swab samples are collected using polyester-tipped plastic swabs. Both swabs are inserted into a single tube containing 3 ml of viral transport medium, which is labeled with a barcode; no personal identifying information is contained on the sample label. For infants <6 months of age, a single oropharyngeal swab is collected. Samples are stored in the Clinical Laboratory at the HCSFV at 4 °C and are transported to the CNDR NVL for processing within 16 h of collection on weekdays and within 48 h on weekends or holidays.

### Data collection, management and security

Numerous tools are implemented to manage the large amounts of data involved with study operation, providing real-time monitoring and facilitating management of screening processes, registration, and long-term follow-up [12]. These tools are highly flexible and secure, and enable compliance with GCP and regulatory guidelines, including US federal regulations (21 CFR 11), via differentiated user roles and privileges, password and user authentication security, and comprehensive auditing to record and monitor access and data changes. The components of the custom informatics system are presented in Additional file 1: Table S1. OpenClinica is the main tool used for data management (OpenClinica LLC, Waltham, MA, https://openclinica.com/). OpenClinica was adapted for the NPICS study and validated to ensure accuracy, reliability, consistent performance, and an audit trail to track invalid/altered records. The system produces accurate paper copies of electronic records via data exports. Backup and restore processes are documented and validated. As many data and lab results as possible are entered automatically through tablets, smart phones, barcodes, and electronic transfer to study databases. When direct entry of data is used, the software uses prompts for out-of-range data and consistent terminology use. Data collection/entry is supervised, and double data entry is used, with multiple “check functions” in place. Alteration of data is strictly controlled.

Data from field visits, the annual survey/sampling, and clinical histories are collected directly into tablet computers or smart phones to allow real-time access to data and to minimize missing fields and data entry mistakes. Applications for data entry are designed with appropriate skip patterns and questions to verify responses. Use of mobile devices with barcode scanners allows access to study databases and the sample tracking system during power outages, when essential study computers are maintained using backup batteries or a small power generator. In the NVL, temperatures of all freezers, refrigerators, and incubators are recorded via smart phones twice daily, and graphical representations of temperature variation are generated. In the HCSFV, mobile devices, GIS and barcodes are all utilized during field visits to participants’ homes [12]. The geographic localization of cohort participants are mapped using GIS, which is particularly useful since Managua lacks addresses and street names. GIS-generated maps are used in daily logistics of planning efficient deployment of field teams [11].

### Tracking samples and forms

Specimens and forms are tracked using a barcode system to minimize data entry error and allow for real-time tracking of all data and samples [10, 12]. Aviles et al. [11] One-dimensional and two-dimensional barcodes are used as appropriate for form or tube size. Barcodes for sample storage at -80 °C or in liquid nitrogen are printed on special labels (Cryocool, Zebra Technologies Corporation, Lincolnshire, IL). Each time a sample or form is scanned, the date and time are automatically recorded. Importantly, barcodes are scanned at each transfer between study personnel and sites and when processes are completed. In the HCSFV, all medical consultation forms are pre-printed with barcodes. Forms that are not used for any reason are recorded in the database as a discarded form to enable rapid identification any missing forms.

### Mobile data collection and Dashboard

A custom informatics system for study fieldwork called Dashboard was developed to improve the speed and completeness of data and sample collection. Users and roles for mobile data applications are configured into the server backend. Before using applications in the phone, all participant information in the dashboard is synchronized with study databases using MySQL odbc driver. With the application and forms installed in smart phones and tablets, a valid user enters into the application in the mobile device, and credentials are checked on the server. If the user is validated, the application synchronizes the data with the server, recording all information into the mobile database; this allows users to work in the field without network connectivity if necessary. In the application, users can search for a participant by barcode or by study code to pull up the participant’s information and enter data. In the application, Dashboard users can see participant information, including household and demographic information, and readily access information on other participants in the household. The application indicates which processes remain to be completed for the participant, thereby increasing data completeness, validates that all required information is entered, and prompts the user to indicate why missing data or samples are not being collected at that time. Information collected on mobile devices through Dashboard include the participant, household, breastfeeding and study satisfaction surveys, height and weight, sample data, field visits, consent data, vaccination information, study gift receipt, supervisor sample reception, and sample temperature data. All information from the mobile application, along with clinical laboratory information, is sent to the server to update the web monitoring dashboard in real-time.

**Fig. 1:**
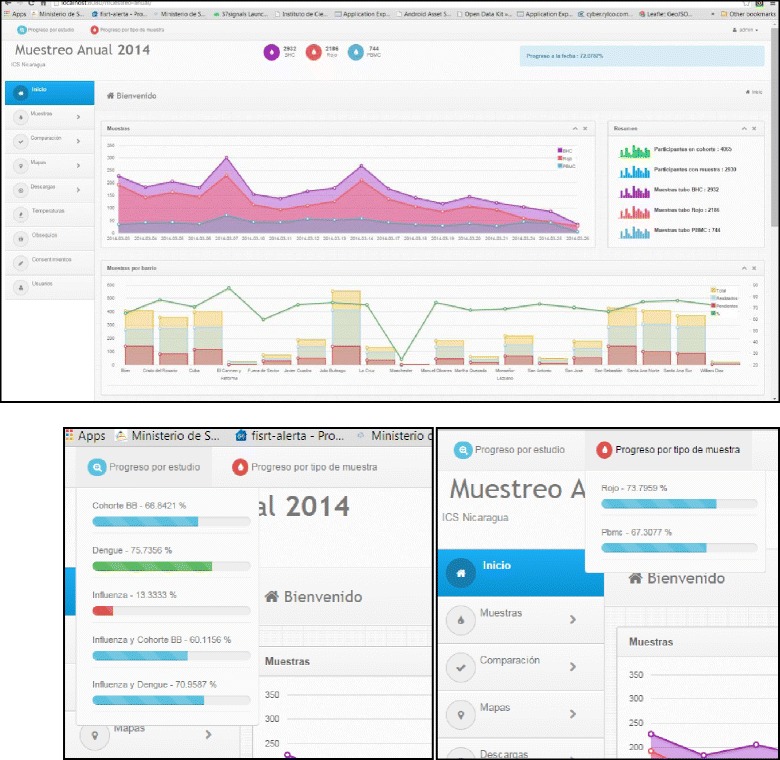
Custom web-based application, Dashboard

**Fig. 2:**
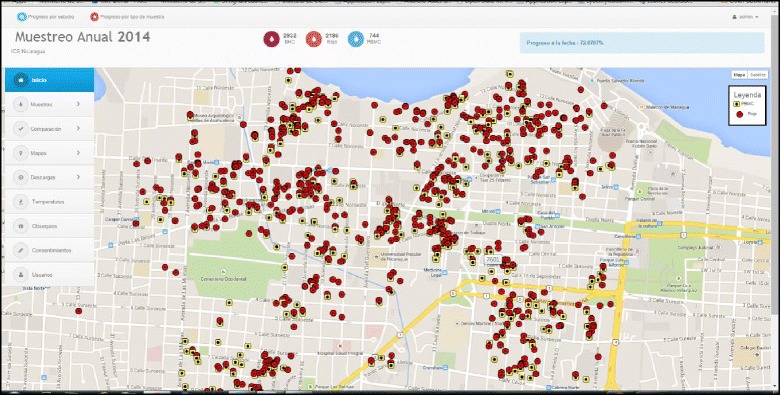
Dashboard map of samples collected during the annual sample collection, 2014

In Dashboard, study personnel can review summary statistics and graphics, such as samples by day, type, neighborhood, and study (Fig. 1). All sample and data information is available in a searchable table. Dashboard has a quality control check built into the application to keep track of samples and shows immediately any discrepancy at any point in the process, for example, if a sample is recorded as collected but is not received by the laboratory. The application also has a mapping function that can present samples collected or samples to be collected in real-time (Fig. 2). This graphical presentation assists supervisors to redirect field staff to maximize productivity of field teams and eliminate duplicated efforts. Finally, users can review information and graphics about temperature, study gift receipt, and consent collected by field teams. The system as a whole allows for quick access to study information to make timely decisions and maximize efficiency, facilitate logistics, and assure data and sample quality.

### Managing multiple studies

In addition to the NPICS, the HCSFV is also the site for 3 other large prospective field studies; a household study of influenza transmission, an influenza birth cohort study, and a Pediatric Dengue Cohort Study (PDCS). Many of the participants are enrolled in more than one study. For instance, there is substantial overlap of participants ≥2 in the NPICS and the PDCS. Studying two diseases in the same population enables more efficient use of resources; however, extra checks must be implemented to prevent protocol violations. Both electronic and visual checks assist study personnel in identifying the studies in which participants are enrolled and in collecting appropriate samples and data. Electronic checks alert personnel that the participant has not completed a consent form, such as the DNA consent, or do not permit a barcode to be printed for a research sample that should not be collected (e.g., a respiratory sample for a child only in the dengue cohort). Additionally, the participants’ ID has a colored band that indicates the study(s) in which the child is participating. Another important feature is that we try minimize the impact of participating in multiple studies on the participant and their family. The consent forms for each study specifically grant permission for participants’ data and samples to be shared between studies, both current and past/future. This enables sharing data and annual samples between studies, minimizing risks and burden for participants.

### Capacity building

A number of initiatives have been conducted to strengthen human capacity at the Ministry of Health sites where the study is conducted. All new study personnel participate in a 2-5-day training program that includes a presentation on GCP, the NIH Human Subjects course, and training in the study protocol and standard operating procedures. Continuing personnel also receive training in relevant areas. Training provided on-site through visits of US-based investigators have included workshops on: biostatistics and STATA, sample collection, phylogenetics and mathematical modeling, and the Responsible Conduct of Research. Several training workshops and seminars were facilitated through FHI360/NIAID: GCP, Data Management, Fundamentals of International Clinical Research, and Clinical Research Design. The study also supported the training of laboratory technicians and two in-country MPH degrees. Additionally, training has been provided to support study personnel in: NIH Program Funding & Grants Administration and electronic Research Administration. Altogether, this broad range of training opportunities has significantly contributed to the current study and has increased the research and administrative capacity at the study sites.

### Laboratory methods

#### RT-PCR

RNA is extracted from nasal and oropharyngeal swabs using the QIAamp® Viral RNA Mini Kit (Qiagen Corporation, Valencia, CA). Influenza viruses A and B are amplified and typed/subtyped according to the protocol of the Centers for Disease Control and Prevention (CDC).

#### Peripheral Blood Mononuclear Cells (PBMCs)

PBMCs are prepared from a subset of annual samples, including repeat influenza virus infections. Briefly, blood samples collected at the HCSFV or in the field are promptly transported to the NVL/CNDR for immediate processing, as described previously [13]. Time-stamps are implemented at all stages from sample collection to freezing to ensure sample integrity. Viability after thawing PBMCs is evaluated on a random selection of samples (90 % viability on average).

## Discussion

### Characteristics of study population and participation

**Fig. 3:**
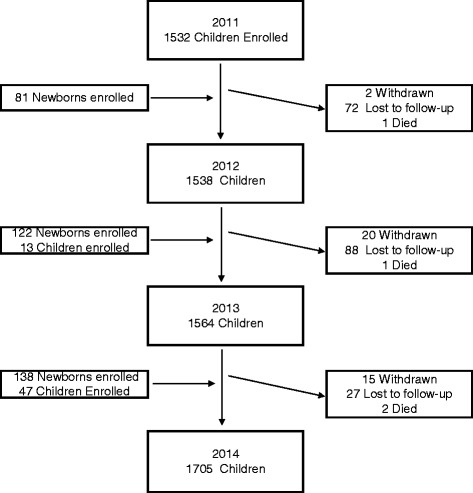
Flow chart of study participation

Between January 1, 2011, and December 31, 2013, 1933 children participated in the NPICS. A flowchart of study participation is presented in Fig. 3, and participant characteristics in Table 2. Among the children, 1532 were enrolled from all age groups between 0 and 12 years of age at the start of the study and subsequently, 401 children were enrolled over the following three years. During the first three years of the study, 268 (13.9 %) children were withdrawn, lost-to-follow-up or died. The mean yearly loss to follow-up was 4.6 % (range 2.5–6.5 %). The main reasons that children were withdrawn (*n* = 37) included that the child had reached the maximum age for the study, study physicians felt it was in the child’s best interest, or the parent no longer wanted the child to contribute respiratory or blood samples. Four children died during the study; all deaths were in children under the age of 1 year.

**Table 2:** Participant characteristics by year, Nicaraguan Pediatric Influenza Cohort Study, Managua, Nicaragua, 2011–2013

	2011	2012	2013
	*n* = 1613	*n* = 1673	*n* = 1748
	No. (%)	No. (%)	No. (%)
By sex			
Female	814 (50.5)	844 (49.4)	884 (50.6)
Male	799 (49.5)	829 (49.6)	864 (49.4)
By age (years)			
0	226 (14.0)	224 (13.4)	252 (14.4)
1	138 (8.6)	138 (8.2)	97 (5.6)
2	104 (6.4)	125(7.5)	129 (7.4)
3	119 (7.4)	97 (5.8)	116 (6.6)
4	108 (6.7)	114 (6.8)	131 (7.0)
5	108 (6.7)	104 (6.2)	108 (6.4)
6	125 (7.8)	104 (6.2)	101 (5.8)
7	123 (7.6)	121 (7.2)	98 (5.6)
8	130 (8.1)	118 (7.0)	116 (6.6)
9	144 (8.9)	123 (7.4)	113 (6.5)
10	150 (9.3)	134 (8.0)	113 (6.5)
11	137 (8.5)	142 (8.5)	122 (7.0)
12	1 (0.1)	129 (7.7)	136 (7.8)
13	-	-	116 (6.6)

Children attended a total of 20,934 medical visits, including 15,794 initial visits and 5140 follow-up visits. A total of 1724 (89.2 %) children attended one or more medical visits. The median number of medical visits per child was 7 (range 0–108). Upper respiratory infections were by far the most common reason for seeking medical care, comprising a total of 9439 (59.8 %) initial visits and 2143 (41.7 %) follow-up appointments. Reasons for seeking medical care are summarized in Table 3. The vast majority (91.6 %) of children with febrile illness presented in the first 4 days since onset of symptoms.

**Table 3:** Reasons for medical visits, Nicaraguan Pediatric Influenza Study, Managua, Nicaragua, 2011–2013

	Initial Medical Visits	Follow-up Visits	Total
	*n* = 15,794	*n* = 5140	*n* = 20,934
	No. (%)	No. (%)	No. (%)
Upper respiratory	9439 (59.8)	2143 (41.7)	11,582 (55.3)
Lower respiratory	300 (1.9)	358 (6.7)	658 (3.1)
Gastrointestinal illness	1777 (11.2)	478 (9.3)	2255 (10.8)
Febrile illness	669 (4.2)	1067 (20.8)	1736 (8.3)
Dermatological	844 (5.3)	123 (2.4)	967 (4.6)
Trauma	435 (2.8)	36 (0.7)	471 (2.3)
Other	2300 (14.8)	935 (18.2)	3265 (15.6)

### Study strengths

Much remains to be elucidated about the epidemiology and transmission of influenza in tropical regions. Here we describe the study design and methods of the Nicaraguan Pediatric Influenza Cohort Study, which was established to examine the incidence and risk factors for influenza infection and disease severity in children and to study the seasonality of influenza in Managua, Nicaragua. We show that loss to follow-up was low, participants accessed medical care regularly, and respiratory illness was the most common reason for seeking medical care. We also describe our methods to increase adherence to study protocols, comply with GCP, and conduct multiple large-scale field studies in a single site using shared infrastructure.

The NPICS evolved from and benefited from several ongoing and past studies at this site, including the Pediatric Dengue Cohort Study and our previous influenza cohort study [8, 10]. Through all of these studies, we were able to engage in significant local capacity building, such as establishing molecular biological, virological, and serological influenza diagnostics in the NVL and training local investigators in field study methodologies, including GCP. In addition, this study and our other studies at the HCSFV serve as enhanced sentinel sites for the Nicaraguan Ministry of Health, with influenza results delivered in real-time to health officials. For instance, during the 2009 pandemic, our ongoing influenza study detected the first case of pandemic influenza in the country, and the government was immediately made aware.

The development of a customized, relatively low-cost informatics system is one of the unique features of this study. The informatics system was designed in-house by the study informatics team not only to increase efficiency and data completeness, but also to enable researchers to minimize protocol deviations. The issue of protocol deviations can become particularly challenging when several protocols are run from the same study site and participants can participate in one or various studies. In particular, the Dashboard developed for the study allows for real-time monitoring of sample collection and helps prevent a child from having their annual sample collected twice. All of the informatics and database system complies with US federal requirements (21 CFR 11).

### Study Limitations

A major limitation of this study is that it relies on passive surveillance, that is the parents must bring the child into the health center for care. The use of passive surveillance will result in the underestimation of influenza rates, particularly among less severe cases. To increase compliance with attendance at the health center we have made study care available 24 h a day, every day of the year and have minimized wait time to see a physician. In addition, we maintain regular contact with participants and their families to encourage attendance for medical care at the Health Center.

## Conclusions

In summary, we have designed and implemented a large community-based influenza cohort study in children in Managua, Nicaragua, with high participation and compliance with study procedures. We have designed a comprehensive, low-cost informatics system to aid in the management of the study, increase data quality, and decrease protocol deviations. The cohort study is ongoing and is continuing to provide essential information on the burden and seasonality of influenza in Nicaraguan children. We also demonstrate the successful integration of two cohort studies (influenza and dengue) in the same site and population, thus maximizing effective use of human, financial, and infrastructural resources.

## Additional file

Additional file 1: Table S1. Components of Custom Informatics System, Nicaraguan Pediatric Influenza Cohort Study, Managua, Nicaragua, 2011-2013. (DOC 46 kb)

## Abbreviations

CNDR: Centro Nacional de Diagnóstico y Referencia
GCP: good clinical practice
HCSFV: Health Center Socrates Flores Vives
NPICS: Nicaraguan Pediatric Influenza Cohort Study
NVL: National Virology Laboratory
