# Supplementary material for: The Nicaraguan pediatric influenza cohort study: design, methods, use of technology, and compliance
Source: BMC Infect Dis. 2015 Nov 9;15:504. doi: 10.1186/s12879-015-1256-6 (PMC4640204; doi:10.1186/s12879-015-1256-6)
Supplement: Additional file 1: Table S1. — Components of Custom Informatics System, Nicaraguan Pediatric Influenza Cohort Study, Managua, Nicaragua, 2011-2013. (DOC 46 kb) [file 12879_2015_1256_MOESM1_ESM.doc]

| Additional file 1 Table 1. Components of Custom Informatics System, Nicaraguan Pediatric Influenza Cohort Study, Managua, Nicaragua, 2011-2013 | | |
| --- | --- | --- |
|  |  |  |
| **Function** |  | **Components** |
|  |  |  |
| Clinical chart |  | OpenClinica Version 3.1.4.1 (https://community.openclinica.com/) |
|  |  | Implemented using apache tomcat 6.029 (http://tomcat.apache.org/), PostgreSQL 9.1 (http://www.postgresql.org/), on a debian 6 server (https://www.debian.org/). |
|  |  | [Reference for requirements and installation: https://docs.openclinica.com/3.1/installation/install-3-1-x-linux](https://docs.openclinica.com/3.1/installation/install-3-1-x-linux) |
|  |  |  |
| Fingerprint Recognition Tool |  | [Verifinger Standard Development Kit (http://www.neurotechnology.com/verifinger.html)](http://www.neurotechnology.com/verifinger.html) |
|  |  | Developed using Microsoft Visual Basic (Microsoft Corporation, Seattle, Washington) |
|  |  | [Scanner: Digital Persona U.are.U 4000B fingerprint scanner (http://www.crossmatch.com/Home/)](http://www.crossmatch.com/Home/) |
|  |  | [Supported scanners: (http://www.neurotechnology.com/cgi-bin/fingerprint-scanners.cgi)](http://www.neurotechnology.com/cgi-bin/fingerprint-scanners.cgi) |
|  |  |  |
| Geographic Information System |  | For field collection of GPS data: Android mobile devices using Open Data Kit Collect (https://opendatakit.org/use/collect/) |
|  |  | [For analysis of geographic information: ArcView 10.1 (http://www.esri.com/software/arcgis/arcgis-for-desktop)](http://www.esri.com/software/arcgis/arcgis-for-desktop) |
|  |  | [Storage of GPS data: Open Data Kit Aggregate (https://opendatakit.org/use/aggregate/)](https://opendatakit.org/use/aggregate/) |
|  |  | [Implemented using apache tomcat 6.029 (http://tomcat.apache.org/), MySQL 5.5](http://tomcat.apache.org/) |
|  |  | (http://dev.mysql.com/doc/refman/5.5/en/index.html), on a debian 6 server (https://www.debian.org/). |
|  |  |  |
| Barcode Printing and Reading |  | Zebra Polypro 1000 labels for labeling paperwork and blood collection tubes. |
|  |  | Zebra Cryocool 3000 labels for labeling Eppendorf tubes and cryovials. |
|  |  | Zebra TLP 2844 thermal transfer printers. |
|  |  | Honeywell MS7580, MS9520, MS9544, Opticon LG2, Opticon 3301 hand-held scanners for scanning barcodes with automatic time stamp and mobile tracking. |
|  |  | Microsoft Access Databases (Microsoft Corporation, Seattle, Washington) |
|  |  | [Jollytech Print Studio Professional (Now LabelFlow http://www.jollytech.com/products/label-flow/index.php )](http://www.jollytech.com/products/label-flow/index.php) |
|  |  |  |
| Mobile Data Collection System |  | [For participant follow up and surveys in the field, a mobile application for Android using Java was developed and used in combination with a modified version of ODK Collect (https://opendatakit.org/use/collect/) , Forms developed using Formhub (https://formhub.org/)](https://opendatakit.org/use/collect/) |
|  |  | Backend dashboard developed using java (https://java.com/en/), spring framework (http://projects.spring.io/spring-framework/) and bootstrap (HTML, CSS, and JS framework for developing responsive, mobile first projects on the web http://getbootstrap.com/ ). |
|  |  | Implemented using apache tomcat 6.029 (http://tomcat.apache.org/), MySQL 5.5 (http://dev.mysql.com/doc/refman/5.5/en/index.html), on a debian 6 server (https://www.debian.org/). |
|  |  |  |
| Barcode-based System for Sample Management |  | Microsoft Access Database application developed in the health center clinical lab. Labels are printed before collection using bidimensional code. |
